# High incidence of RAS pathway mutations among sentinel genetic lesions of Korean pediatric *BCR‐ABL1*‐like acute lymphoblastic leukemia

**DOI:** 10.1002/cam4.3099

**Published:** 2020-05-07

**Authors:** Jae Wook Lee, Yonggoo Kim, Bin Cho, Seongkoo Kim, Pil‐Sang Jang, Jaewoong Lee, Hanwool Cho, Gun Dong Lee, Nack‐Gyun Chung, Myungshin Kim

**Affiliations:** ^1^ Department of Pediatrics College of Medicine The Catholic University of Korea Seoul Republic of Korea; ^2^ Department of Laboratory Medicine College of Medicine The Catholic University of Korea Seoul Republic of Korea; ^3^ Catholic Genetic Laboratory Center College of Medicine Seoul St. Mary’s Hospital College of Medicine, The Catholic University of Korea Seoul Republic of Korea

**Keywords:** acute lymphoblastic leukemia, *BCR-ABL1*-like, RAS mutation

## Abstract

**Introduction:**

Recent advances in genetic analysis have led to the discovery of novel genetic subtypes of precursor B‐cell acute lymphoblastic leukemia (B‐ALL) with prognostic relevance. In this study, we studied a cohort of pediatric B‐ALL patients to retrospectively determine the incidence of patients harboring novel genetic subtypes, as well as their outcome.

**Methods:**

B‐ALL patients (N = 190) diagnosed in a single Korean hospital were included in the study. Patients' medical records were reviewed for data on established genetic abnormalities and outcome. *CRLF2* expression was analyzed by quantitative RT‐PCR. Anchored multiplex PCR‐based enrichment was used to detect fusions and point mutations in 81 ALL‐related genes.

**Results:**

Incidence of established recurrent genetic subtypes was as follows: high hyperdiploidy (21.6%), *ETV6‐RUNX1* (21.6%), *BCR‐ABL1* (7.9%), *KMT2A* rearrangement (7.4%) *TCF3‐PBX1*/*TCF3‐HLF* (7.4%), and hypodiploidy (1.1%). Incidence of new genetic subtypes was as follows: *BCR‐ABL1*‐like (13.2%), *ETV6‐RUNX1*‐like (2.1%), *EWSR1‐ZNF384* (1.1%), and iAMP21 (1.1%). Median age at diagnosis of *BCR‐ABL1*‐like ALL was 6.8 years. According to type of genetic abnormality, *BCR‐ABL1*‐like ALL was divided into ABL class (12%), CRLF2 class (8%), JAK‐STAT class (12%), and RAS class (68%). The 5‐year event‐free survival (EFS) of *BCR‐ABL1*‐like patients was significantly inferior to non‐*BCR‐ABL1*‐like low‐ and standard‐risk patients (71.5 ± 9.1% vs 92.5 ± 3.2%, *P* = .001) and comparable to non‐*BCR‐ABL1*‐like high (75.2 ± 6.2%) and very high‐risk patients (56.8 ± 7.4%). All four *ETV6‐RUNX1*‐like patients survived event‐free.

**Conclusion:**

Analogous to previous studies, incidence of *BCR‐ABL1*‐like ALL in our cohort was 13.2% with outcome comparable to high and very high‐risk patients. A significantly high number of RAS class mutations was a distinct feature of our *BCR‐ABL1*‐like ALL group.

## Graphical abstract

Incidence of *BCR‐ABL1*‐like ALL in a Korean pediatric cohort was 13.2% with outcome comparable to high and very high‐risk patients. A significantly high number of RAS class mutations was a feature that uniquely identified our *BCR‐ABL1*‐like ALL group.

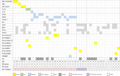

## 1 INTRODUCTION

Recurrent genetic abnormalities in precursor B‐cell acute lymphoblastic leukemia (B‐ALL) are used to define disease subtype and predict patient prognosis. Studies aiming to identify genetic lesions in B‐ALL patients without established genetic abnormalities led to the discovery of *BCR‐ABL1*‐like ALL. Initial studies defined this subtype as having a gene expression profile similar to *BCR‐ABL1* (+) ALL, a high incidence of *IKZF1* deletions, and overall poor prognosis.^1^, ^2^ Subsequent studies showed that the majority of *BCR‐ABL1*‐like ALL patients harbor kinase‐activating abnormalities, including ABL‐class fusions, *CRLF2* rearrangements, *JAK2* fusions, other mutations activating the JAK‐STAT pathway, and mutations of the RAS pathway.^3^, ^4^, ^5^, ^6^ In addition, recent studies identified new genetic subtypes, including those characterized by rearrangements of *DUX4*, *MEF2D*, and *ZNF384*.^7^, ^8^, ^9^

Identification of these newly proposed subtypes is important not only because the subtypes correlate with clinical parameters including outcomes, but also because they reveal therapeutic targets that may allow a reduction in morbidity and mortality related to conventional chemotherapy. However, optimum diagnostic strategy of these new genetic subtypes in the clinic lacks consensus, and several practical considerations remain.

In this study, we determined the genetic characteristics, including the incidence of patients harboring new genetic subtypes, as well as outcome in a cohort of pediatric B‐ALL treated at a single Korean hospital. In addition, we tried to outline the optimum laboratory methodology required for accurate identification of new genetic subtypes.

## 2 PATIENTS AND METHODS

### 2.1 Study group

Patients diagnosed with B‐ALL and treated at the Department of Pediatrics in Seoul St. Mary's Hospital from March 2009 to September 2015 were included in the study (n = 190, male 116 (61.1%)). The study received institutional review board approval from Seoul St. Mary's Hospital, The Catholic University of Korea (IRB No. KC17SESI0717). Diagnosis of ALL was done according to the WHO Classification of Tumours of Haematopoietic and Lymphoid Tissues based on bone marrow (BM) pathology, immunophenotyping, cytogenetics, and molecular genetics.^10^ Presence of established, recurrent genetic abnormalities of pediatric B‐ALL (*ETV6‐RUNX1*, *BCR‐ABL1*, *E2A‐PBX1, KMT2A* rearrangements, high hyperdiploidy, hypodiploidy) in the patients was diagnosed using reverse transcription polymerase chain reaction (RT‐PCR), fluorescence in situ hybridization (FISH) and Giemsa band karyotyping, using methods reported previously.^11^ High hyperdiploidy was defined as a karyotype with 51‐65 chromosomes, while hypodiploidy was diagnosed with <45 chromosomes. All patients were risk classified and treated according to an institutional protocol, the details of which have been reported previously.^12^ Clinical data, including age and initial white blood cell (WBC) count at diagnosis, National Cancer Institute/Rome (NCI) risk group,^13^ and institutional risk group^12^ were recorded.

### 2.2 Targeted next‐generation sequencing based on anchored multiplex PCR

From the overall study group, 127 patients were classified as B‐ALL with the six recurrent genetic abnormalities (Table 1). High hyperdiploidy and *ETV6‐RUNX1* were most common, with both detected in 41 patients (21.6%). *BCR‐ABL1* was detected in 15 patients (7.9%), followed by *KMT2A* rearrangement (n = 14, 7.4%), *TCF3‐PBX1*/*TCF3‐HLF* (n = 14, 7.4%), and hypodiploidy (n = 2, 1.1%). Among 63 patients without recurrent genetic abnormalities, 49 patients with remaining samples underwent further genetic analysis including anchored multiplex PCR (AMP) (Figure S1). First, we measured *CRLF2* gene expression by quantitative RT‐PCR (RT‐qPCR) as described in a previous report.^14^ The relative expression levels were estimated using the 2^−ΔΔCt^ method. Then, AMP was performed to determine gene expression, fusions, exon‐skipping and point mutations associated with the *BCR‐ABL1*‐like subtype using the Archer FusionPlex ALL kit (ArcherDX). Briefly, reverse transcription using random primers was performed for synthesis of cDNA, followed by end repair and adenylation steps. The cleanup of cDNA using Agencourt^®^ AMPure^®^ XP beads and ligation of molecular barcode (MBC) adapters and universal primer sites were performed. The MBC adapter‐attached cDNA was amplified by the GSP1 primer pool and primer complementary to universal primer site, and the second PCR using GSP2 primer pool was performed. The libraries were quantitated using a KAPA Universal Library Quantification Kit (Kapa Biosystems), then normalized and loaded to NextSeq (Illumina). Data were analyzed by Archer^®^ Analysis version 5.1.7 (ArcherDX).

**TABLE 1:** Genetic classification of the study group

	190 (%)
Recurrent genetic abnormalities	127 (66.8)
High hyperdiploidy	41 (21.6)
*ETV6‐RUNX1*	41 (21.6)
*BCR‐ABL1*	15 (7.9)
*KMT2A* rearrangement	14 (7.4)
*TCF3‐PBX1*	14 (7.4)^a^
Hypodiploidy	2 (1.1)
Other	63 (33.2)^b^
iAMP21	2 (1.1)
*BCR‐ABL1*‐like ALL	25 (13.2)
*ETV6‐RUNX1*‐like ALL	4 (2.1)
*EWSR1‐ZNF384*	2 (1.1)
B‐other	16 (8.4)

Abbreviation; iAMP21, intrachromosomal amplification of chromosome 21.

^a^ Including one *TCF3‐HLF*.

^b^ Including 14 cases with suboptimal RNA.

### 2.3 Confirmatory tests for gene fusion by RT‐PCR and FISH

RT‐PCR and FISH were performed to confirm the fusion genes detected by AMP NGS. RT‐PCR was carried out using primers that were previously described in other studies and designed by Primer3 (http://bioinfo.ut.ee/primer3‐0.4.0/).^4^ Amplification conditions were as follows; initial denaturation at 95°C for 5 minutes, 25 PCR cycles, denaturation at 95°C for 30 seconds, annealing at 58°C for 30 seconds, extension at 72°C for 90 seconds and hold at 4°C.

In addition, we underwent FISH as an alternative method to identify gene fusions, using *ABL1* break‐apart probe (Cytocell Ltd), *JAK2* break‐apart probe (Cytocell Ltd), *IGH* break‐apart probe (Cytocell Ltd) and *P2RY8* deletion probe (Cytocell Ltd).

### 2.4 Confirmatory tests for gene mutation by massive parallel sequencing

We validated genetic mutations identified from AMP NGS with those from massive parallel sequencing. DNA was extracted from patients’ BM aspirates using Wizard^®^ Genomic DNA Purification kit (Promega). We used a clinically targeted panel (Customized St. Mary's hematology NGS panel; ThermoFisher Scientific) and the Ion Torrent Sequencer, S5XL. Base calling and alignment of the sequences to reference genome hg19 were performed on the Ion Torrent Suite Software (Version 5.8.0). Variant calling was done using Ion Reporter software (Version 5.6). Elaborated sequence data in FASTQ format were adjusted and annotated according to the hg19 human reference genome.

### 2.5 Study objectives

The initial objective of the study was to retrospectively determine the incidence of established genetic abnormalities, as well as novel genetic subtypes such as *BCR‐ABL1*‐like ALL in our study group. The subsequent objective was to analyze clinical parameters at diagnosis and EFS of patients with novel genetic subtypes.

### 2.6 Statistical analysis

Comparisons of median age and WBC count at diagnosis, and ALL risk group classification between genetic subgroups was done with the Mann‐Whitney test and Pearson's chi‐square test, respectively. Patients with RAS class mutations comprised a significant subgroup of *BCR‐ABL1*‐like ALL patients. Clinical and treatment response‐based parameters predicting the presence of RAS class mutation were analyzed by logistic regression. EFS and OS were determined by Kaplan‐Meier method, with comparisons of EFS done with log‐rank test. EFS was defined as the time from diagnosis to last follow‐up in CR, or first event (relapse, primary refractory disease, death, or secondary malignancy). OS was defined as the time from diagnosis to last follow‐up or death from any cause. The date of last follow‐up was 31 September 2019. *P* value < .05 was considered significant.

## 3 RESULTS

### 3.1 Genetic classification of the study group

**FIGURE 1:**
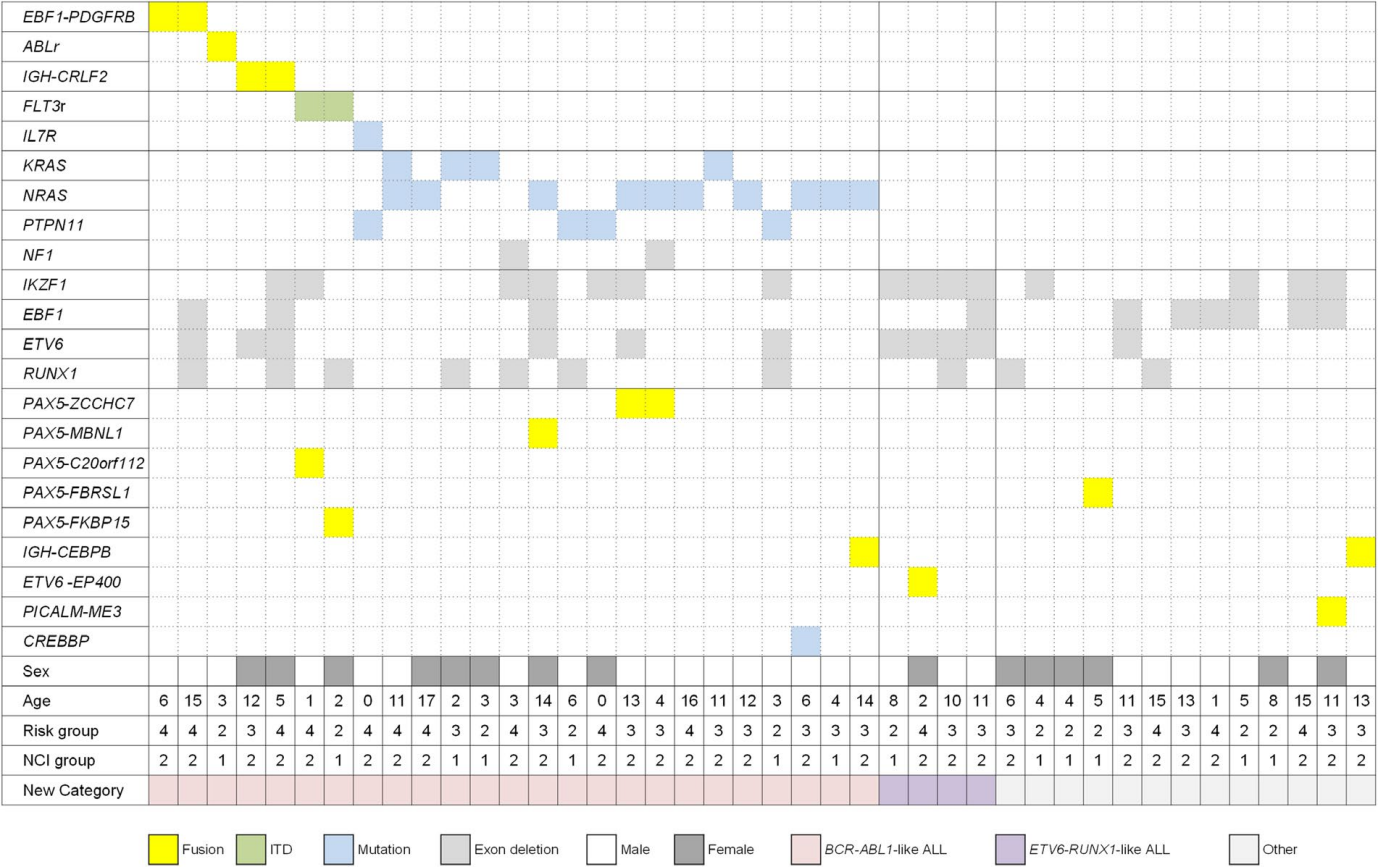
Genetic and clinical characteristics of patients. Data are shown for 25 patients with *BCR‐ABL1*‐like ALL, 4 with *ETV6‐RUNX1*‐like ALL and 13 other B‐ALL. *ABL1r*, *ABL1* rearrangement; *FLT3r*, *FLT3* rearrangement; NCI, National Cancer Institute/Rome

Among 49 patients who lacked established recurrent genetic abnormalities, we identified two patients with intrachromosomal amplification of chromosome 21 (iAMP 21) by FISH. Gene fusion was detected in 18 patients; *RANBP2‐ABL1* (n = 1), *EBF1‐PDGFRB* (n = 2), *IGH‐CRLF2* (n = 2), other *IGH* rearrangement (n = 2), *EWSR1‐ZNF384* (n = 2), *PAX5* rearrangement (n = 6), *TCF3* rearrangement (n = 1), *PICALM‐ME3* (n = 1), and *ETV6‐EP400* (n = 1) (Figure 1).

We also detected single nucleotide variations and small insertion/deletions by FusionPlex^®^ ALL. Mutations activating JAK‐STAT pathways were identified in three patients including *IL7R* (n = 1) and *FLT3* (n = 2). Interestingly, RAS pathway mutations were most commonly identified (n = 17); *NRAS* (n = 8), *KRAS* (n = 3), *KRAS* and *NRAS* (n = 1), *NRAS* and *NF1* (n = 1), *PTPN11* (n = 3), and *NF1* (n = 1) (Table S1). For the three patients with *PTPN11* mutations, genetic study of complete remission BM samples was negative, indicating that the mutations were somatic status. Clinical and treatment response‐based factors (patient gender, age at diagnosis, initial WBC count, NCI risk group, prephase steroid response of peripheral blasts) did not significantly predict the presence of RAS mutations (data not shown). Exon skipping was also detected in *ETV6* (n = 6), *RUNX1* (n = 7), *EBF1* (n = 3), and *IKZF1* (n = 7).

We grouped patients carrying genetic abnormalities associated with *BCR‐ABL1*‐like ALL including ABL‐class fusions, *CRLF2* rearrangements, JAK‐STAT pathway mutations and RAS pathway mutations, and classified them as *BCR‐ABL1*‐like ALL (n = 25, 13.2% of B‐ALL) (Table 2). We classified patients as *ETV6‐RUNX1*‐like ALL when both *ETV6* and *IKZF1* rearrangements were identified (n = 4, 2.1% of B‐ALL). The remaining 16 patients (8.4%) were grouped as B‐other ALL (Table 1).

**TABLE 2:** Sentinel genetic lesions of 25 *BCR‐ABL1*‐like ALL

Genetic lesions	No (%)
ABL‐class fusions	3 (12)
*EBF1‐PDGFRB*	2 (8)
*RANBP2‐ABL1*	1 (4)
CRLF2 rearrangements	2 (8)
*IGH‐CRLF2*	2 (8)
JAK‐STAT pathway mutations	3 (12)
*IL7R* mutation	1 (4)
*FLT3* mutation	2 (8)
RAS pathway mutations	17 (68)
*NRAS*	8 (32)
*KRAS*	3 (12)
*NRAS and KRAS*	1 (4)
*NRAS and NF1*	1 (4)
*PTPN11*	3 (12)
*NF1*	1 (4)

### 3.2 Gene expression of the study group

We compared gene expression between *BCR‐ABL1*‐like ALL, *ETV6‐RUNX1*‐like ALL and other B‐ALL. The expression of the *CRLF2*, *PDGFRB* and *IRF8* genes was higher in *BCR‐ABL1*‐like ALL, while expression of the *SH2B3*, *NTRK3*, *SOX11* genes was lower in *BCR‐ABL1*‐like ALL (Figure S2). Then, we analyzed and compared gene expression within the *BCR‐ABL1*‐like ALL group of patients, according to type of genetic abnormality. Because each group consisted of a limited number of patients, there was no significant difference in gene expression among the patients. *CRLF2* expression was highest in patients with *CRLF2* rearrangement. In patients with ABL‐class rearrangement, the *SEMA6A* and *EBF1* genes showed high expression (Figure S3).

### 3.3 Clinical characteristics and outcome of new subgroups

The median age at diagnosis of the *BCR‐ABL1*‐like ALL subgroup was 6.1 years (range: 0.6‐18), and did not differ significantly from that of the non‐*BCR‐ABL1*‐like ALL subgroup. The median WBC count of the *BCR‐ABL1*‐like ALL subgroup was 18.98 × 10^9^/L (range: 0.89‐161.61 × 10^9^/L), and again did not show any clear difference from that of the non‐*BCR‐ABL1*‐like ALL subgroup. A significantly higher proportion of *BCR‐ABL1*‐like ALL patients were classified as NCI high risk than non‐*BCR‐ABL1*‐like ALL patients. Also, a greater proportion of patients was classified as either high or very high‐risk according to institutional classification criteria among *BCR‐ABL1*‐like ALL patients than among non‐*BCR‐ABL1*‐like ALL patients (Table 3).

**TABLE 3:** Comparison of clinical characteristics of the *BCR‐ABL1*‐like ALL and non‐*BCR*‐*ABL1*‐like ALL subgroups

	*BCR‐ABL1*‐like ALL (N = 25)	Non‐*BCR‐ABL1*‐like ALL (N = 165)	*P* value
Median age at diagnosis (range)	6.1 y (0.6‐18.0)	5.4 y (0.2‐17.1)	0.311
Median WBC count at diagnosis (range)	18.98 × 10^9^/L (0.89‐161.61)	12.70 × 10^9^/L (1.21‐726.93)	0.332
NCI risk group (%)			0.011
Standard	7 (28)	92 (56)	
High	18 (72)	73 (44)	
Overall risk group (%)^b^, ^a^			0.033
Low	0 (0)	44 (27)	
Standard	5 (20)	24 (15)	
High	10 (40)	49 (30)	
Very high	10 (40)	48 (29)	

Abbreviations: NCI, National Cancer Institute; WBC, white blood cell.

^a^ Based on institutional risk group criteria.

The 5‐year EFS of the *BCR‐ABL1*‐like ALL subgroup was 71.5 ± 9.1% (18/25). There was no significant difference in EFS and OS for *BCR‐ABL1*‐like ALL patients with or without RAS pathway mutations (Figure S4). Events included six patients with relapsed disease and one patient who died of acute respiratory distress syndrome during remission induction chemotherapy. Median time to event was 21.5 months (range: 0.9‐46 months). Three of the six relapsed patients currently survive disease‐free, two patients after receiving chemotherapy and allogeneic hematopoietic cell transplantation (HCT) and one patient after receiving chemotherapy and scheduled for allogeneic HCT, resulting in a 5‐year OS for the *BCR‐ABL1*‐like ALL subgroup of 83.6 ± 7.5% (21/25).

Two of the six *BCR‐ABL1*‐like ALL patients who relapsed were standard risk according to institutional criteria, with the remainder being high or very high‐risk. Hence, for the standard‐risk group as a whole (N = 29), all of the 24 non‐*BCR‐ABL1*‐like ALL standard‐risk patients survive event‐free, while two of the five *BCR‐ABL1*‐like ALL patients experienced an event (5‐year EFS of 100% vs 53.3%, *P* < .001).

**FIGURE 2:**
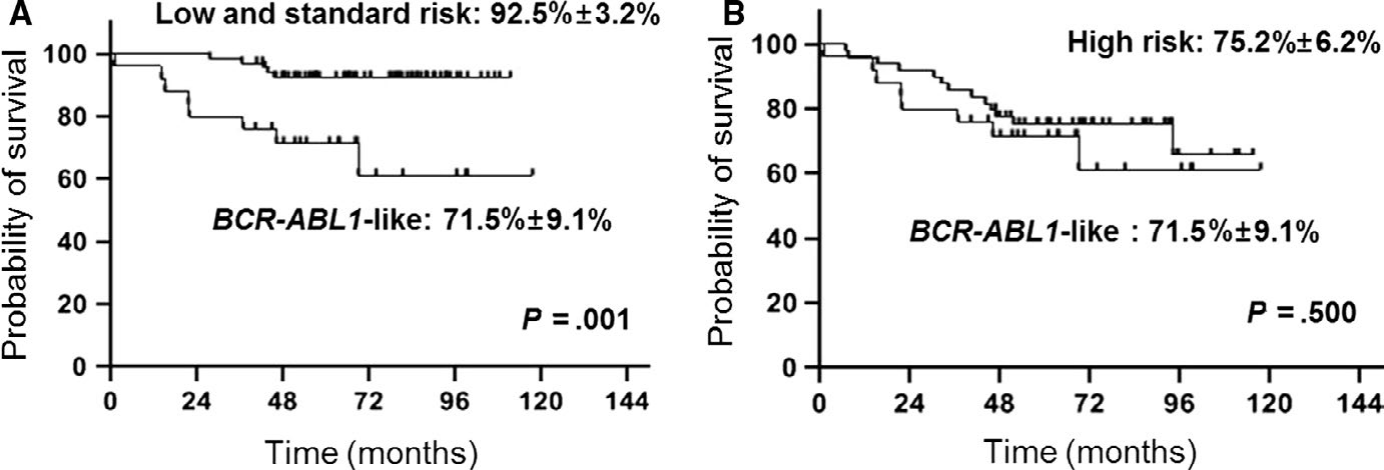
Comparison of 5‐y EFS of *BCR‐ABL1*‐like ALL subgroup and non‐*BCR‐ABL1*‐like low and standard risk subgroup (A), and non‐*BCR‐ABL1*‐like high risk subgroup (B)

The 5‐year EFS of the *BCR‐ABL1*‐like ALL subgroup was significantly lower than that of the non‐*BCR‐ABL1*‐like ALL low‐ and standard‐risk patients (92.5 ± 3.2%, *P* = .001), and was similar to the 5‐year EFS of 75.2 ± 6.2% found for non‐*BCR‐ABL1*‐like ALL high‐risk patients (Figure 2). Also, no significant difference was found in the 5‐year EFS between *BCR‐ABL1*‐like ALL patients and non‐*BCR‐ABL1*‐like ALL very high‐risk patients (56.8 ± 7.4%, *P* = .367).

*ETV6‐RUNX1*‐like patients did not show significant difference in clinical characteristics including age and WBC count compared with other B‐ALL patients (data not shown). All 6 *ETV6‐RUNX1*‐like patients survived event‐free.

## 4 DISCUSSION

Recent advances in genetic analysis have led to the identification of novel genetic subtypes of B‐ALL with prognostic relevance. In this study, we studied a cohort of pediatric B‐ALL patients to retrospectively determine the incidence of patients harboring new genetic subtypes, as well as their outcome. We successfully identified patients in the *BCR‐ABL1*‐like ALL category by detecting gene fusions and mutations. *BCR‐ABL1*‐like ALL comprised 13.2% of pediatric B‐ALL in our cohort, an incidence similar to that reported by previous studies.^2^, ^15^ A key distinguishing feature of *BCR‐ABL1*‐like ALL is aberrant signaling through cytokine and tyrosine kinase receptors.^4^ Common genetic abnormalities include rearrangements of *CRLF2*, ABL‐class tyrosine kinase genes, and *JAK2*, as well as mutations activating JAK‐STAT and RAS signaling.

RAS pathway mutations had the highest frequency in our *BCR‐ABL1*‐like ALL patients (N = 17). Previous studies showed that patients with RAS mutations do not comprise a significant proportion of *BCR‐ABL1*‐like ALL patients, with incidence ranging from 3.6% to 6%.^4^, ^5^, ^16^, ^17^ In contrast, another study showed that *BCR‐ABL1*‐like ALL is one of the subtypes of ALL in which RAS mutations are more prevalent, with an incidence of clonal RAS mutations reported in approximately 30% of *BCR‐ABL1*‐like ALL patients.^18^ Several studies of RAS mutation in Asian children with ALL showed a higher incidence than had been reported in patients of Western nations.^19^, ^20^ Similarly, patients with RAS mutations may form a significant subset of Asian patients with *BCR‐ABL1*‐like ALL. Recent studies based on Asian patients focused on the identification of kinase fusions, *IKZF1* deletions, and *JAK* mutations associated with *BCR‐ABL1*‐like ALL.^21^, ^22^ Hence, further studies are necessary to ascertain potential ethnic differences in the role of RAS mutation in *BCR‐ABL1*‐like ALL.

In terms of clinical parameters, pediatric patients with *BCR‐ABL1*‐like ALL are likely to be older and have a high WBC count at diagnosis, resulting in a greater proportion of NCI high‐risk patients in the *BCR‐ABL1*‐like ALL category.^5^ These clinical features, as well as the greater likelihood of having high levels of minimal residual disease after induction chemotherapy than other B‐ALL patients,^23^ are key factors contributing to the overall poor outcome of *BCR‐ABL1*‐like ALL patients. In our study, a significantly higher proportion of *BCR‐ABL1*‐like ALL patients were classified as high risk according to both NCI and institutional classification criteria compared with non‐*BCR‐ABL1*‐like ALL patients.

The 5‐year EFS of the *BCR‐ABL1*‐like ALL subgroup was significantly lower than that of the non‐*BCR‐ABL1*‐like ALL low‐ and standard‐risk patients, and was similar to that of non‐*BCR‐ABL1*‐like ALL high‐risk patients. Inferior outcome of *BCR‐ABL1*‐like ALL patients compared with low‐ and standard‐risk patients is consistent with the majority of *BCR‐ABL1*‐like ALL patients being high risk according to both NCI and institutional criteria. However, two of the five standard‐risk *BCR‐ABL1*‐like ALL patients relapsed, while all of the 24 non‐*BCR‐ABL1*‐like ALL standard‐risk patients survive event‐free. One extensive study showed that standard‐risk *BCR‐ABL1*‐like ALL patients had significantly worse EFS than other standard‐risk patients,^17^ indicating that the *BCR‐ABL1*‐like ALL genotype may influence the role of well‐established prognostic factors in pediatric ALL.

Gene expression profiling is the main method for diagnosis of *BCR‐ABL1*‐like ALL. However, it is not widely available in routine clinical practice. Several studies using an 8‐ or 15‐gene quantitative assay using TaqMan‐based low‐density array showed high correlation with gene expression profiling,^5^, ^16^ but the low‐density array is only available in a few clinical laboratories.^24^ In this study, we screened *BCR‐ABL1*‐like ALL using AMP NGS. This method utilizes specific and universal primers, allowing for diagnosis of a translocation without the identification of both fusion partners.^25^ When gene rearrangements and mutations were detected, the sentinel genetic abnormalities of *BCR‐ABL1*‐like ALL were confirmed by RT‐PCR, FISH or sequencing. Through this strategy, we identified sentinel gene fusions that may be used as therapeutic targets. A substantial number of clinical reports have shown that patients with kinase‐activating lesions associated with *BCR‐ABL1*‐like ALL are amenable to targeted therapy, with tyrosine kinase inhibitors for patients with ABL‐class fusions, and JAK inhibitors for those with *JAK2* fusions or mutations.^26^, ^27^, ^28^, ^29^, ^30^ Identification of sentinel genetic abnormalities associated with *BCR‐ABL1*‐like ALL may allow for novel and effective targeted therapy. Other recurrent genetic abnormalities including *ETV6*‐*RUNX1*‐like ALL and *ZNF384* rearrangements were identified in this study. Although past studies have tried to characterize their incidence and clinical significance,^9^, ^15^ prognostic implications of these novel B‐ALL subtypes require further study.

In summary, our cohort of *BCR‐ABL1*‐like ALL patients comprised 13% of B‐ALL, had inferior outcome compared with low‐ and standard‐risk patients, and had a high incidence of RAS class mutations. This is the first report to demonstrate the characteristics of *BCR‐ABL1*‐like ALL in Korean pediatric patients. Further analysis based on a greater number of *BCR‐ABL1*‐like ALL patients is necessary to confirm the key findings of our study.

## CONFLICT OF INTEREST

The authors declare that they have no competing interests.

## AUTHOR CONTRIBUTIONS

Conceptualization and design: YK, N‐GC, and MK. Patient data and samples: JWL, BC, SK, and P‐SJ. Experiments, collection and assembly of data: JL, HC, and GDL. Data analysis and interpretation: JWL, YK, HC, and MK. Manuscript writing and editing: JWL, N‐GC, YK, and MK.

## Supporting information

Supinfo

## Data Availability

The data that support the findings of this study are available from the corresponding author upon reasonable request.
