# Supplementary material for: High incidence of RAS pathway mutations among sentinel genetic lesions of Korean pediatric BCR‐ABL1‐like acute lymphoblastic leukemia
Source: Cancer Med. 2020 May 7;9(13):4632–9. doi: 10.1002/cam4.3099 (PMC7333828; doi:10.1002/cam4.3099)
Supplement: Supplementary file 1 — Supinfo [file CAM4-9-4632-s001.pptx]

## Slide 1
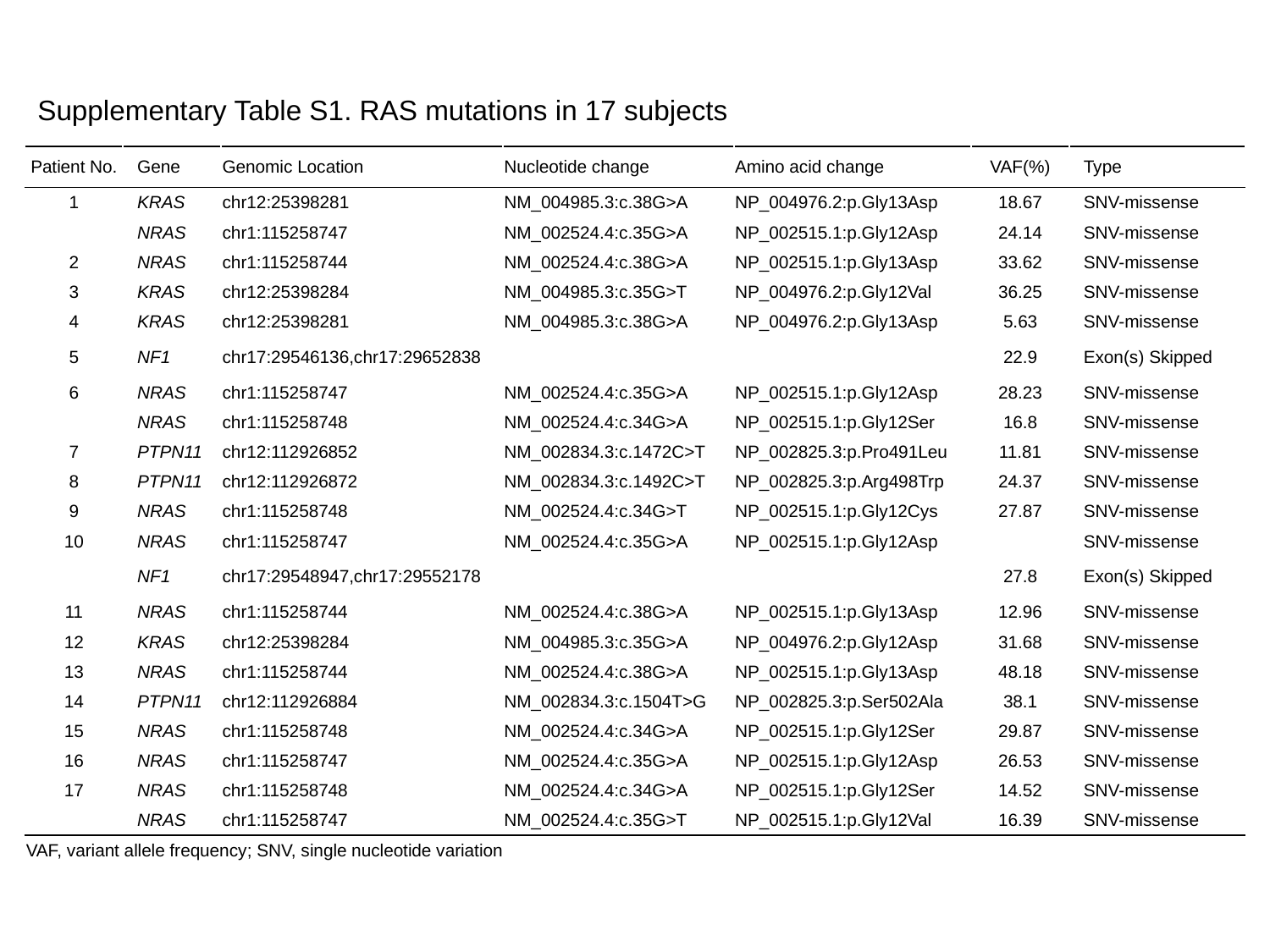

Supplementary Table S1. RAS mutations in 17 subjects
| Patient No. | Gene | Genomic Location | Nucleotide change | Amino acid change | VAF(%) | Type |
| --- | --- | --- | --- | --- | --- | --- |
| 1 | KRAS | chr12:25398281 | NM\_004985.3:c.38G>A | NP\_004976.2:p.Gly13Asp | 18.67 | SNV-missense |
| | NRAS | chr1:115258747 | NM\_002524.4:c.35G>A | NP\_002515.1:p.Gly12Asp | 24.14 | SNV-missense |
| 2 | NRAS | chr1:115258744 | NM\_002524.4:c.38G>A | NP\_002515.1:p.Gly13Asp | 33.62 | SNV-missense |
| 3 | KRAS | chr12:25398284 | NM\_004985.3:c.35G>T | NP\_004976.2:p.Gly12Val | 36.25 | SNV-missense |
| 4 | KRAS | chr12:25398281 | NM\_004985.3:c.38G>A | NP\_004976.2:p.Gly13Asp | 5.63 | SNV-missense |
| 5 | NF1 | chr17:29546136,chr17:29652838 | | | 22.9 | Exon(s) Skipped |
| 6 | NRAS | chr1:115258747 | NM\_002524.4:c.35G>A | NP\_002515.1:p.Gly12Asp | 28.23 | SNV-missense |
| | NRAS | chr1:115258748 | NM\_002524.4:c.34G>A | NP\_002515.1:p.Gly12Ser | 16.8 | SNV-missense |
| 7 | PTPN11 | chr12:112926852 | NM\_002834.3:c.1472C>T | NP\_002825.3:p.Pro491Leu | 11.81 | SNV-missense |
| 8 | PTPN11 | chr12:112926872 | NM\_002834.3:c.1492C>T | NP\_002825.3:p.Arg498Trp | 24.37 | SNV-missense |
| 9 | NRAS | chr1:115258748 | NM\_002524.4:c.34G>T | NP\_002515.1:p.Gly12Cys | 27.87 | SNV-missense |
| 10 | NRAS | chr1:115258747 | NM\_002524.4:c.35G>A | NP\_002515.1:p.Gly12Asp | | SNV-missense |
| | NF1 | chr17:29548947,chr17:29552178 | | | 27.8 | Exon(s) Skipped |
| 11 | NRAS | chr1:115258744 | NM\_002524.4:c.38G>A | NP\_002515.1:p.Gly13Asp | 12.96 | SNV-missense |
| 12 | KRAS | chr12:25398284 | NM\_004985.3:c.35G>A | NP\_004976.2:p.Gly12Asp | 31.68 | SNV-missense |
| 13 | NRAS | chr1:115258744 | NM\_002524.4:c.38G>A | NP\_002515.1:p.Gly13Asp | 48.18 | SNV-missense |
| 14 | PTPN11 | chr12:112926884 | NM\_002834.3:c.1504T>G | NP\_002825.3:p.Ser502Ala | 38.1 | SNV-missense |
| 15 | NRAS | chr1:115258748 | NM\_002524.4:c.34G>A | NP\_002515.1:p.Gly12Ser | 29.87 | SNV-missense |
| 16 | NRAS | chr1:115258747 | NM\_002524.4:c.35G>A | NP\_002515.1:p.Gly12Asp | 26.53 | SNV-missense |
| 17 | NRAS | chr1:115258748 | NM\_002524.4:c.34G>A | NP\_002515.1:p.Gly12Ser | 14.52 | SNV-missense |
| | NRAS | chr1:115258747 | NM\_002524.4:c.35G>T | NP\_002515.1:p.Gly12Val | 16.39 | SNV-missense |
| VAF, variant allele frequency; SNV, single nucleotide variation | | | | | | |

## Slide 2
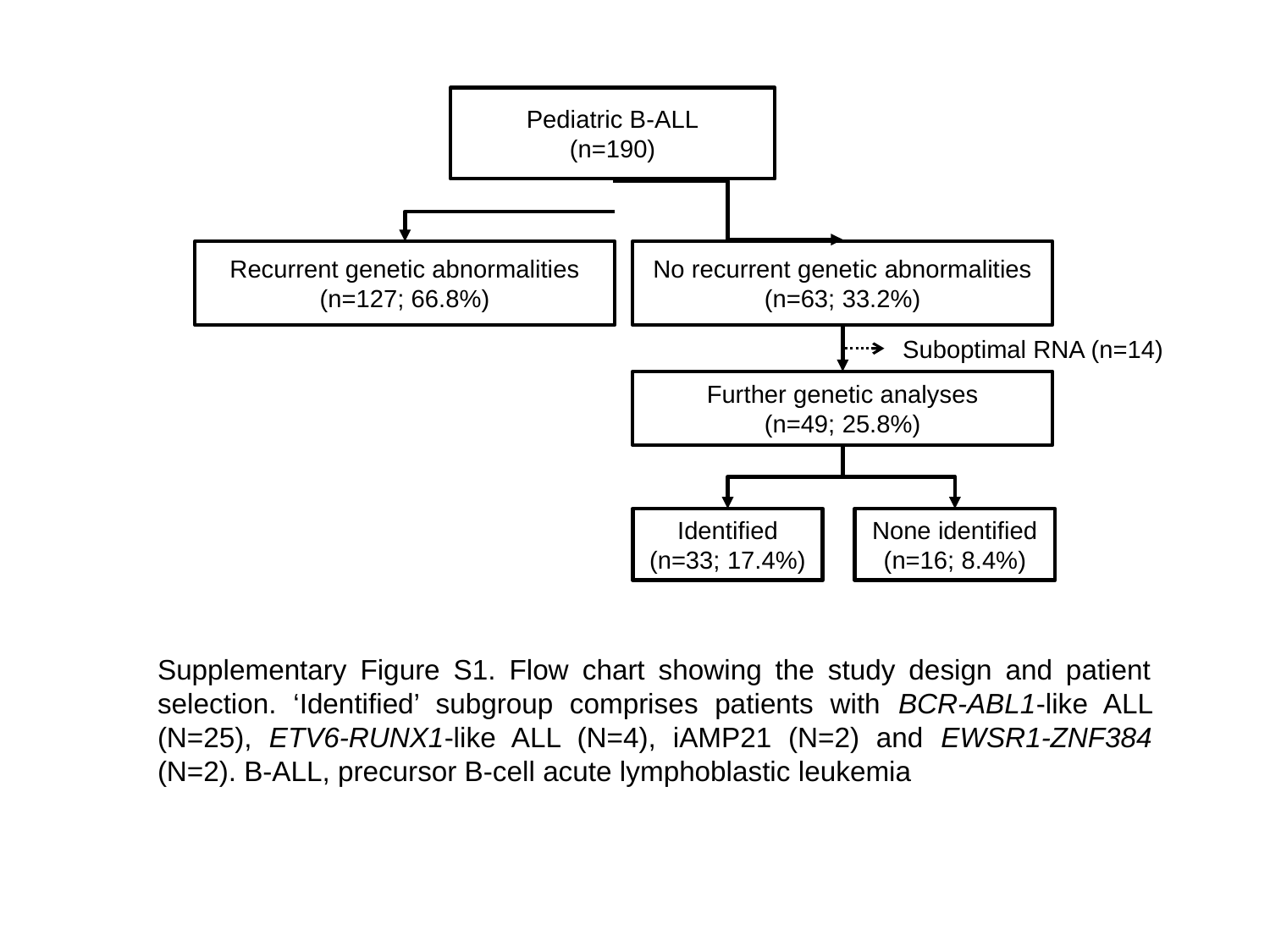

Pediatric B-ALL
(n=190)
No recurrent genetic abnormalities
(n=63; 33.2%)
Recurrent genetic abnormalities
(n=127; 66.8%)
Suboptimal RNA (n=14)
Further genetic analyses
(n=49; 25.8%)
None identified
(n=16; 8.4%)
Identified
(n=33; 17.4%)
Supplementary Figure S1. Flow chart showing the study design and patient selection. ‘Identified’ subgroup comprises patients with BCR-ABL1-like ALL (N=25), ETV6-RUNX1-like ALL (N=4), iAMP21 (N=2) and EWSR1-ZNF384 (N=2). B-ALL, precursor B-cell acute lymphoblastic leukemia

## Slide 3
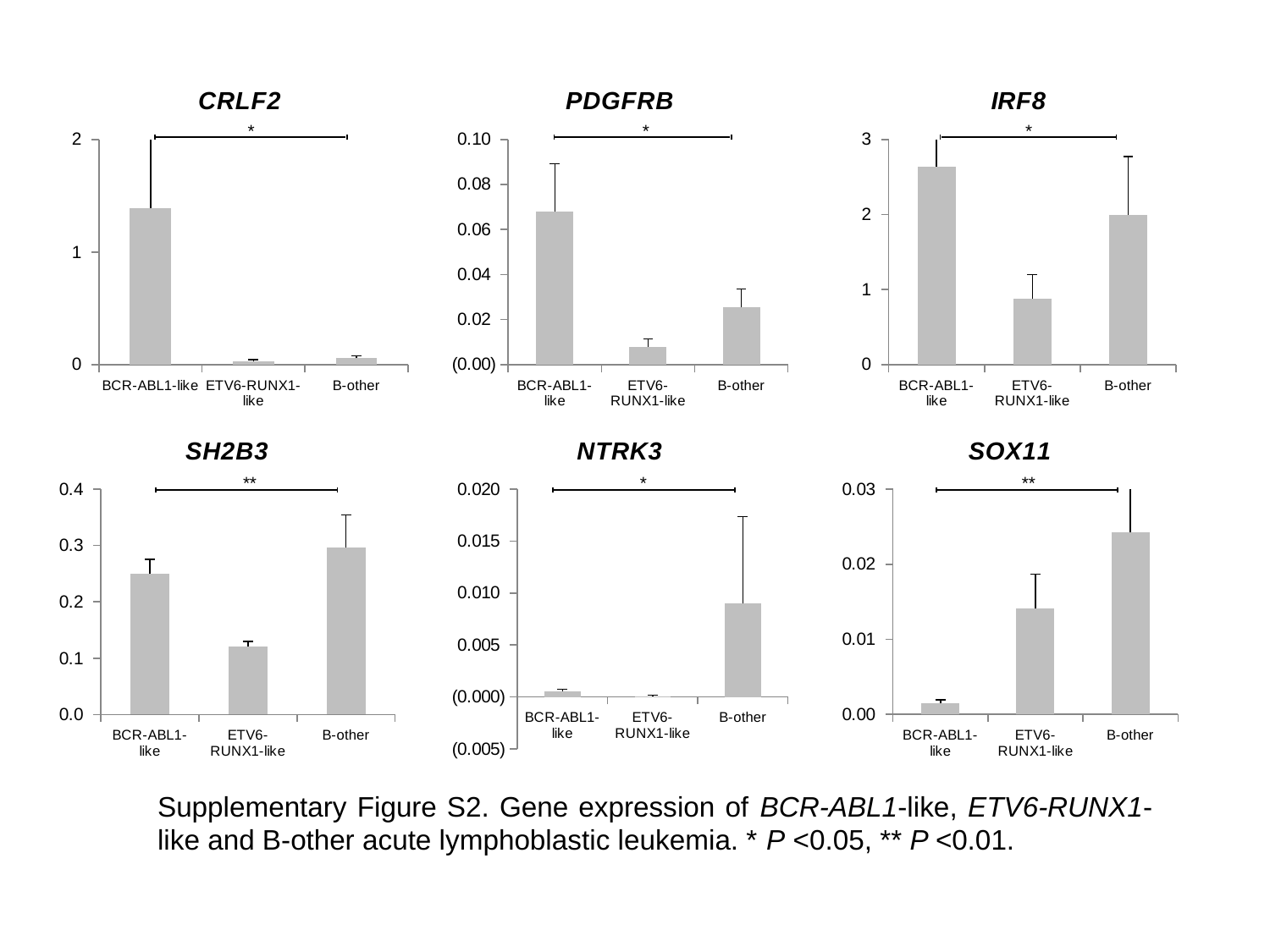

### Chart:
| Category | CRLF2 |
|---|---|
| BCR-ABL1-like | 1.3884559884600005 |
| ETV6-RUNX1-like | 0.025274812562500037 |
| B-other | 0.0554954760769231 |
### Chart:
| Category | PDGFRB |
|---|---|
| BCR-ABL1-like | 0.0678617309028572 |
| ETV6-RUNX1-like | 0.007833489821428579 |
| B-other | 0.025357507945054952 |
### Chart: IRF8
| Category | IRF8 |
|---|---|
| BCR-ABL1-like | 2.6409188336933336 |
| ETV6-RUNX1-like | 0.8759929812083334 |
| B-other | 1.9926022413461548 |*
*
*
### Chart:
| Category | SH2B3 |
|---|---|
| BCR-ABL1-like | 0.2494543194800002 |
| ETV6-RUNX1-like | 0.12029937900000005 |
| B-other | 0.2960656198461538 |
### Chart:
| Category | NTRK3 |
|---|---|
| BCR-ABL1-like | 0.0005412335249999999 |
| ETV6-RUNX1-like | 7.718431250000005e-05 |
| B-other | 0.008984206403846158 |
### Chart:
| Category | SOX11 |
|---|---|
| BCR-ABL1-like | 0.001436907440000001 |
| ETV6-RUNX1-like | 0.014079261749999994 |
| B-other | 0.02424019946153849 |**
*
**
Supplementary Figure S2. Gene expression of BCR-ABL1-like, ETV6-RUNX1-like and B-other acute lymphoblastic leukemia. * P <0.05, ** P <0.01.

## Slide 4
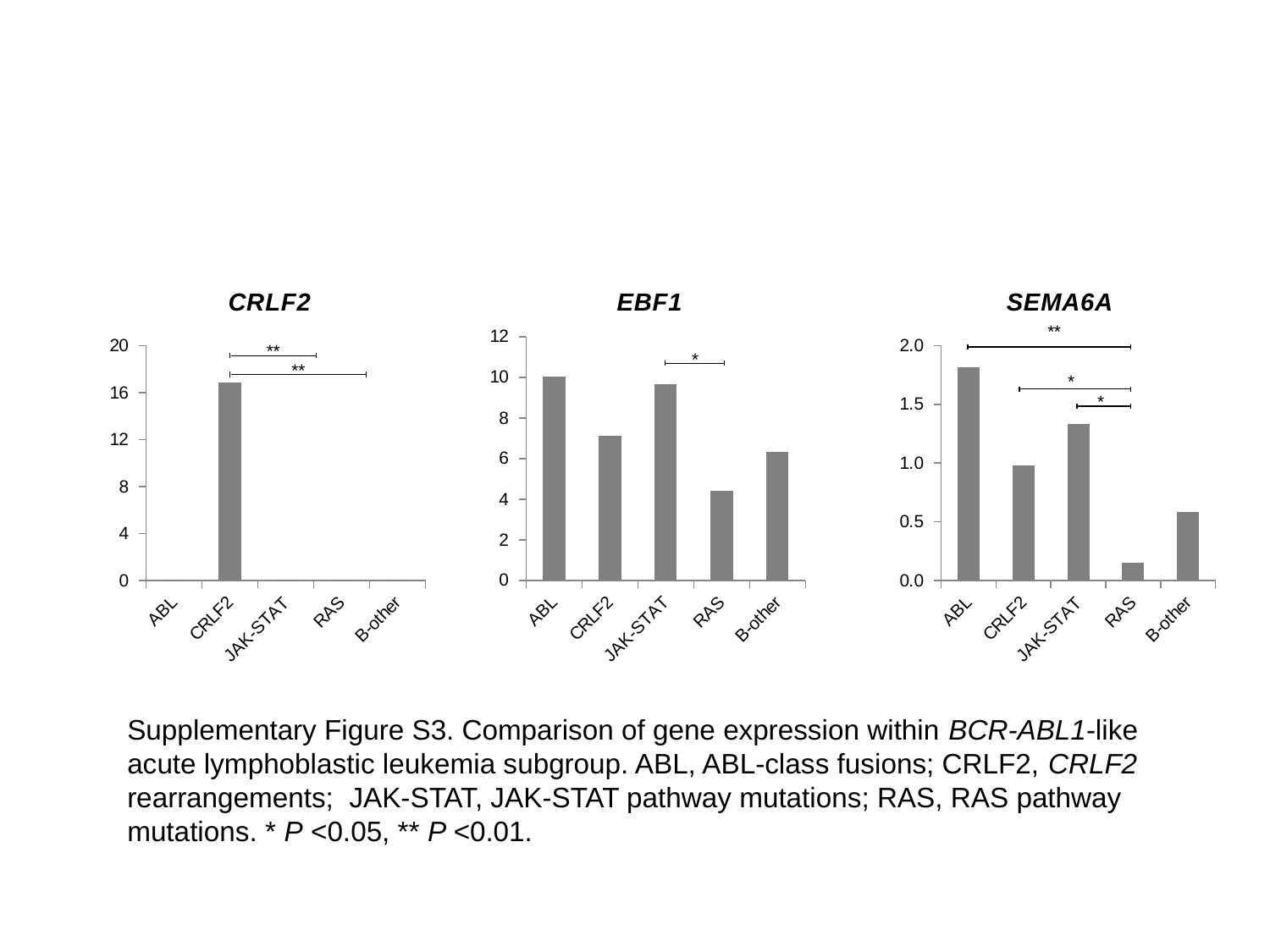

### Chart:
| Category | EBF1 |
|---|---|
| ABL | 10.018179125222218 |
| CRLF2 | 7.119308263666667 |
| JAK-STAT | 9.652898264166675 |
| RAS | 4.424217825019608 |
| B-other | 6.34926009848414 |*
### Chart:
| Category | SEMA6A |
|---|---|
| ABL | 1.814865534166668 |
| CRLF2 | 0.9780678414999999 |
| JAK-STAT | 1.3295791281666665 |
| RAS | 0.15172108041176496 |
| B-other | 0.5810294515714286 |
### Chart
| Category |
|---|
### Chart:
| Category | CRLF2 |
|---|---|
| ABL | 0.08830644195833348 |
| CRLF2 | 16.833499326812518 |
| JAK-STAT | 0.00955515616666669 |
| RAS | 0.0441656625588236 |
| B-other | 0.09276279986309521 |**
**
Supplementary Figure S3. Comparison of gene expression within BCR-ABL1-like acute lymphoblastic leukemia subgroup. ABL, ABL-class fusions; CRLF2, CRLF2 rearrangements; JAK-STAT, JAK-STAT pathway mutations; RAS, RAS pathway mutations. * P <0.05, ** P <0.01.

## Slide 5
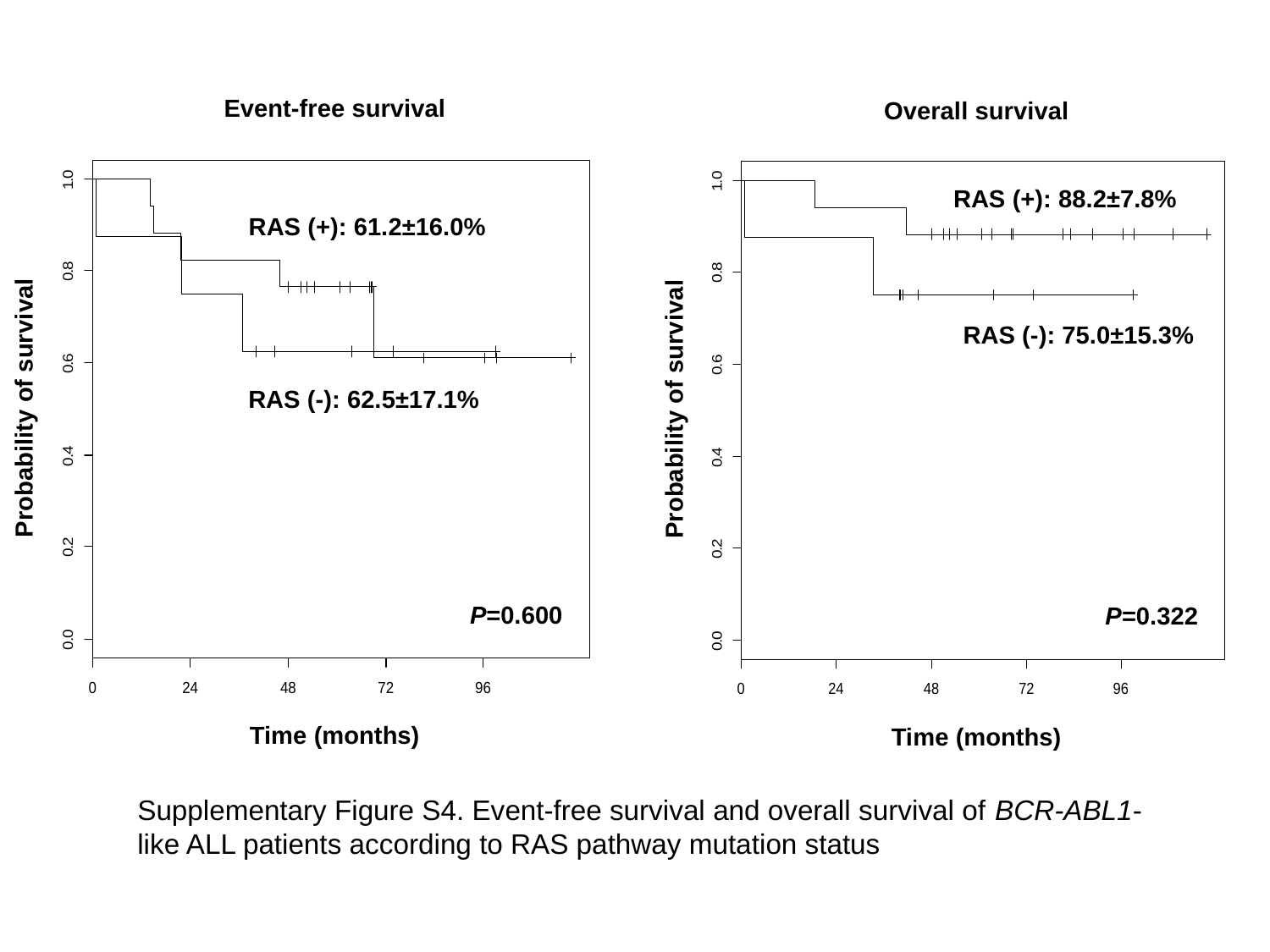

RAS (+): 61.2±16.0%
Probability of survival
Time (months)
RAS (-): 62.5±17.1%
P=0.600
Event-free survival
RAS (+): 88.2±7.8%
Probability of survival
Time (months)
RAS (-): 75.0±15.3%
P=0.322
Overall survival
Supplementary Figure S4. Event-free survival and overall survival of BCR-ABL1-like ALL patients according to RAS pathway mutation status
